# Supplementary figures and images for: Nor1 and Mitophagy: An Insight into Sertoli Cell Function Regulating Spermatogenesis Using a Transgenic Rat Model
Source: Int J Mol Sci. 2025 Sep 20;26(18):9209. doi: 10.3390/ijms26189209 (PMC12471025; doi:10.3390/ijms26189209)

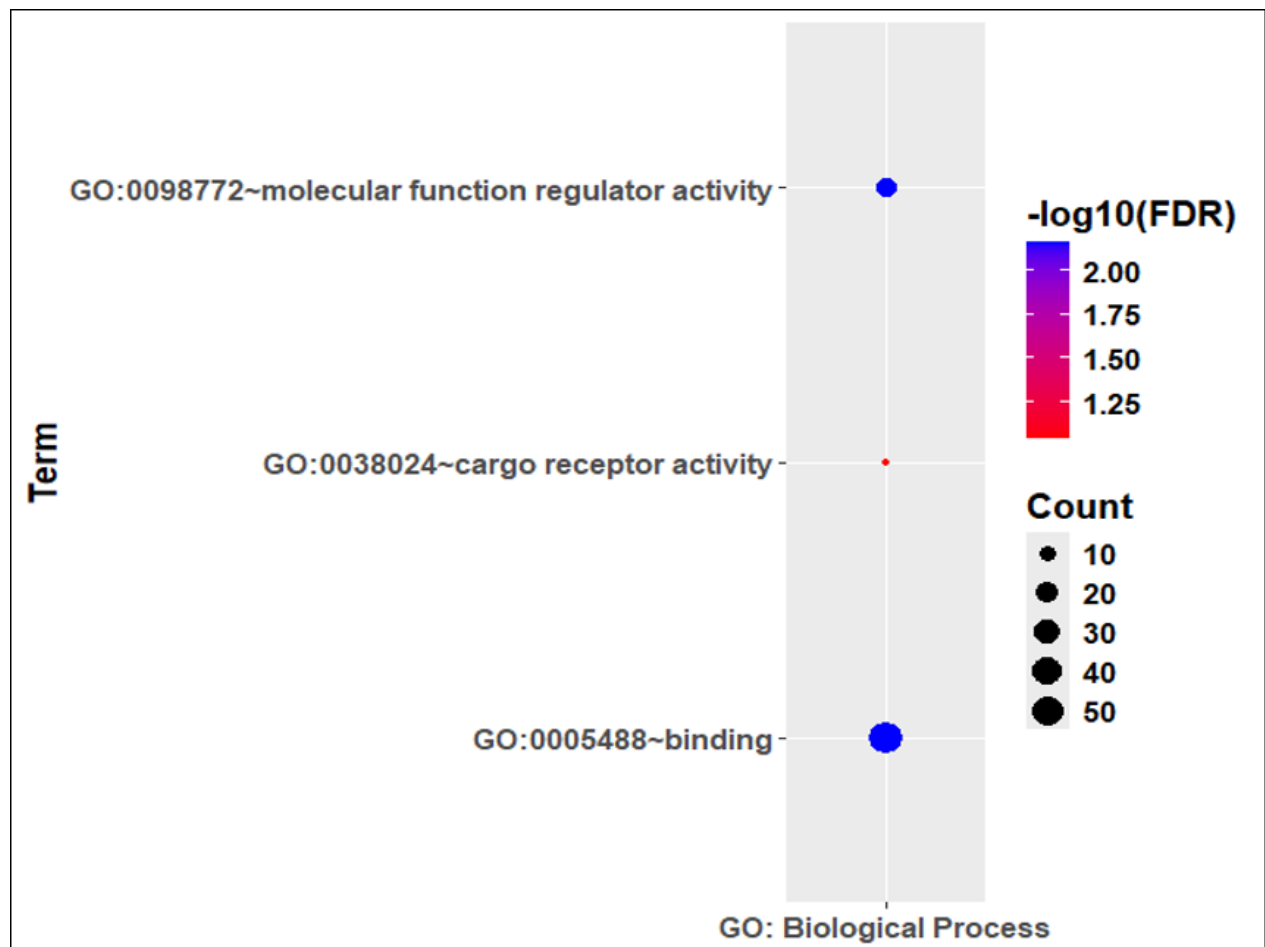

**Supplementary Figure 1:** GO Molecular Function Enrichment of 66 common MRDEGs

Supplement: Supplementary file 1 [file ijms-26-09209-s001.zip › Supplementary Figure 1.pdf]
